# HDAC1 Regulates Neuronal Differentiation

**DOI:** 10.3389/fnmol.2021.815808

**Published:** 2022-01-12

**Authors:** Vanesa Nieto-Estevez, Gopakumar Changarathil, Adebayo Olukayode Adeyeye, Marissa Olga Coppin, Rawan Serena Kassim, Jingfei Zhu, Jenny Hsieh

**Affiliations:** ^1^Department of Neuroscience, Developmental and Regenerative Biology, The University of Texas at San Antonio, San Antonio, TX, United States; ^2^Brain Health Consortium, The University of Texas at San Antonio, San Antonio, TX, United States; ^3^Department of Molecular Biology, UT Southwestern Medical Center, Dallas, TX, United States

**Keywords:** HDAC1, hippocampal neurogenesis, neural stem cells, valproic acid, neuronal differentiation

## Abstract

In adult hippocampal neurogenesis, chromatin modification plays an important role in neural stem cell self-renewal and differentiation by regulating the expression of multiple genes. Histone deacetylases (HDACs), which remove acetyl groups from histones, create a non-permissive chromatin that prevents transcription of genes involved in adult neurogenesis. HDAC inhibitors have been shown to promote adult neurogenesis and have also been used to treat nervous system disorders, such as epilepsy. However, most HDAC inhibitors are not specific and may have other targets. Therefore, it is important to decipher the role of individual HDACs in adult hippocampal neurogenesis. HDACs 1, 2, and 3 have been found expressed at different cellular stages during neurogenesis. Conditional deletion of HDAC2 in neural stem cells impairs neuronal differentiation in adult hippocampus. HDAC3 supports proliferation of adult hippocampal neural stem/progenitor cells. The role of HDAC1 in adult neurogenesis remains still open. Here, we used a conditional knock-out mouse to block HDAC1 expression in neural stem cells (Nestin^+^ cells) during hippocampal neurogenesis. Our results showed that both HDAC1 and HDAC2 are expressed in all cellular stages during hippocampal neurogenesis. Moreover, we found that deletion of HDAC1 by viral infection of neural stem cells is sufficient to compromise neuronal differentiation *in vitro*. However, we were unable to reduce the expression of HDAC1 *in vivo* using Nestin-Cre^ERT2^ mice. Understanding the role of HDAC1 may lead to ways to control stem cell proliferation and neuronal regeneration in the adult hippocampus, and to more specific HDAC therapeutics for neurological disorders.

## Introduction

Chromatin modifications have been shown to be critical during brain development by regulating gene expression in a precise spatial and temporal manner. One such modification is acetylation in the N-terminal tails of histones H3 and H4, which reduces the affinity of the histones to the DNA, opens the chromatin and promotes gene expression. On the other hand, histone deacetylases (HDACs) remove acetyl groups from histones and prevent the transcription of genes (D’Mello, 2020). There are eighteen HDACs, classified into four groups: Class I contains HDACs 1, 2, 3, and 8, which are ubiquitously expressed; class II contains HDACs 4–7, 9, and 10, which are expressed in a tissue-specific manner; Class III also called sirtuins; and Class IV comprises only HDAC11. HDACs form complexes with DNA-binding proteins to interact with the DNA and play critical roles during neurodevelopment. HDAC inhibitors have been used to treat neurological disorders, such as epilepsy, even during pregnancy, although they play key roles during brain formation among other tissues. For instance, valproic acid (VPA) is prescribed in pregnant women to control seizure activity, although prenatal exposure to VPA increases seizure susceptibility in adult offspring due to aberrant hippocampal neurogenesis (Sakai et al., 2018). Moreover, VPA has been shown to promote both neurogenesis and gliogenesis (Hsieh et al., 2004; Lee et al., 2016). The limitation of these HDAC inhibitors is the lack of specificity in their actions affecting multiple genes including non-histone proteins (Milazzo et al., 2020). Some efforts have been made to decipher the role of individual HDACs in neurogenesis.

During embryonic development, HDAC1 and HDAC2 are highly expressed in neuroepithelial cells and neural stem cells (NSCs), and their expression pattern overlaps (Tang et al., 2019). Postnatally, HDAC1 is expressed mainly in astrocytes, whereas HDAC2 is found in neurons (MacDonald and Roskams, 2008; Jawerka et al., 2010). In the adult hippocampus (HP), one of the two regions where neurogenesis persists throughout life, HDAC1 is highly expressed in NSCs and its expression is reduced or undetected in intermediate progenitor cells and neuroblasts, whereas HDAC2 expression is higher in neuroblasts (Jawerka et al., 2010; Foti et al., 2013). The highly conserved amino acid identity of HDAC1 and HDAC2, in addition to their overlapping expression, suggests a redundant role of both HDACs during brain development. In fact, deletion of HDAC1 or HDAC2 alone has no clear phenotype in the brain, whereas the depletion of both leads to severe brain abnormalities (Montgomery et al., 2009; Hagelkruys et al., 2014). In the adult HP, HDAC2 deletion in NSCs leads to a reduction of newly-formed neurons (Jawerka et al., 2010). Moreover, HDAC3 is expressed in progenitor cells and in granule cell neurons in the adult HP. HDAC3 deletion promotes a reduction of neural stem cell proliferation and neuronal differentiation in the adult HP (Jiang and Hsieh, 2014). However, the role of HDAC1 in hippocampal neurogenesis is still unclear.

Here, we first analyzed the expression of HDAC1 and HDAC2 at different stages during adult hippocampal neurogenesis. We found that both HDAC1 and HDAC2 are expressed in all the cellular stages analyzed, although a higher percentage of HDAC1^+^ cells were stem/progenitor cells (Nestin^+^, Sox2^+^ and Ki67^+^) compared to HDAC2^+^ cells, with comparable expression of HDAC1 and HDAC2 in DCX^+^ cells. Because of the higher expression of HDAC1 in progenitor cells, we then isolated NSCs from 6-week old HDAC1^+/+^ and HDAC1^fl/fl^ mice. Once the hippocampal neural stem cells (HPSCs) were expanded, we induced HDAC1 deletion by adenovirus expressing CRE and analyzed cell proliferation by immunostaining and flow cytometry. We found a significant reduction of HDAC1 expression, without affecting HDAC2 expression, but we did not detect any difference in cell proliferation. Next, we induced neural differentiation after HDAC1 deletion. Under differentiation conditions, we found that HDAC1 deletion was associated with impaired neuronal differentiation. Finally, we knocked-out HDAC1 *in vivo* NSCs (Nestin^+^) and we studied its consequences in proliferation (12 days post injection, dpi) and differentiation (28 dpi). Although we detected expression of YFP after Nestin-Cre^ERT2^-mediated recombination of the reporter, we were surprisingly not able to reduce the expression of HDAC1 *in vivo*. Our results showed HDAC1 is necessary for hippocampal neuronal differentiation *in vitro*, but more analysis is needed to clarify the *in vivo* role of HDAC1 during adult hippocampal neurogenesis, and to find more specific HDAC treatments for neurological diseases.

## Materials and Methods

### Mice

All mouse experimental procedures were approved and carried out in accordance with the Institutional Animal Care and Use Committee at the University of Texas Southwestern Medical Center (UTSW) and the University of Texas at San Antonio (UTSA). Mice were housed in an animal facility with 12-h-light/dark cycle, temperature 18–23°C and 40–60% humidity; food and water were administered *ad libitum*. *HDAC1* conditional knock-out (cKO) were obtained by crossing Nestin-Cre^ERT2^, Rosa26-YFP and HDAC1 floxed mice. Nestin-Cre^ERT2^ mice and Rosa26-YFP mice were obtained from the Jackson laboratory, and HDAC1 floxed mice from Dr. Eric Olson (UTSW). Their genotypes were determined by PCR on tail DNA as described previously (Montgomery et al., 2007, 2009; Jiang and Hsieh, 2014). HDAC1 mice were maintained by crossing *HDAC1*^+/fl^ mice. *HDAC1*^+/+^ and *HDAC1*^fl/fl^ littermates were used for experiments. Similarly, HDAC1 cKO were maintained by crossing *HDAC1*^+/fl^*^:^*Nestin-CRE^ERT2^ (hemizygous):ROSA26-YFP (homozygous) mice. To induce the expression of Cre, 6 week-old *HDAC1*^+/+^ and *HDAC1*^fl/fl^:Nestin-Cre^ERT2^:YFP littermates were injected with tamoxifen [TAM (Sigma, Cat. No. BP168-167), 100 mg/kg dissolved in 10% (vol/vol) ethanol and 90% (vol/vol) sunflower oil (Sigma, Cat. No. S5007)] daily for 5 days and then sacrificed at 12 and 28 days after the last day of injection (dpi) for analysis. The numbers of mice used in each experiment are indicated in the figure legends.

Nestin-GFP mice were obtained from the Jackson laboratory (Cat. No. 03392) and used to study the expression of HDAC1 and HDAC2 in NSCs.

### Immunohistochemistry

Mice were perfused intracardially with 4% paraformaldehyde (PFA). Brains were post-fixed in PFA for 24 h, cryoprotected in 30% sucrose for two nights. Serial coronal sections (30 μm) were obtained using a microtome. The sections were first blocked in 0.3% Triton X-100/3% Normal Goat Serum (NGS)/Tris Buffered Saline (TBS) for 1 h and then incubated for 48–72 h at 4°C with the following primary antibodies: guinea pig anti-DCX (1:2,000; Millipore, No. AB2253), chicken anti-GFP (1:500; AvesLab, Cat. No. GFP-1010), rabbit anti-HDAC1 (1:1,000; Abcam, Cat. No. ab19845), rabbit anti-HDAC1 (1:100; Cell Signaling, Cat. No. 34589S), rabbit anti-HDAC2 (1:1,000; Abcam, Cat. No. ab32117), rat anti-Ki67 (1:500; Thermo Fisher Scientific, Cat. No. 14-5698-82), rabbit anti-Pax6 (1:300; Biolegend, Cat. No. 901301), rabbit anti-Prox1 (1:1,000; Millipore, Cat. No. AB5475), rabbit anti-Sox2 (1:1,000; Millipore, Cat. No. AB5603). Sections were then incubated with FITC, Cy3 and Cy5 conjugated secondary antibodies (1:500; Jackson ImmunoResearch). The sections were counterstained with 4′,6-diamidino-2-phenylindole DAPI; Sigma, Cat. No. D9542), then mounted in polyvinyl alcohol solution (PVA; Sigma, Cat. No. BP168-122).

### Quantitative Analysis of Immunostained Sections

Images were taken using a Leica confocal microscope (TCS SPE8). The numbers of HDAC1^+^, HDAC2^+^, Sox2^+^, Ki67^+^, Pax6^+^, DCX^+^, and Prox1^+^ cells were quantified in confocal images of single optical planes taken every 3 μm along the thickness of the sections. The percentage of cells expressing each marker was calculated from the number of YFP^+^ cells.

We used Image J software to analyze the mean signal intensity of HDAC1 staining in YFP^+^ cells. The signal intensity was measured and averaged from more than 100 random individual cells.

To determine the percentage of cells expressing HDAC1 or HDAC2 in wild-type mice, five random images per section were taken in a confocal microscope for all the markers, but two random images per section for Prox1. Five to seven sections were analyzed per mouse.

### Neural Stem Cell Cultures

Hippocampal neural stem cells were isolated from 6-week-old *HDAC1*^+/+^ and *HDAC1*^fl/fl^ mice as described previously (Vergano-Vera et al., 2009; Nieto-Estevez et al., 2016). Briefly, the HP was dissected out of the mouse, cut into small pieces, and digested with 1 mg/ml papain, 0.2 mg/ml cysteine and 0.2 mg/ml EDTA, then gently disaggregated. The resulting cell suspension from each mouse were plated into a well of a 6-multiwell plate. The cells were cultured in Dulbecco’s modified Eagle medium (DMEM)/nutrient mixture F12 (F12), supplemented with insulin (final concentration 10 μg/ml), apotransferrin, putrescine, progesterone, sodium selenite (N2; DMEM/F12/N2) and maintained with daily addition of 20 ng/ml FGF-2 (Peprotech Cat No. 100-18B) and 20 ng/ml EGF (Peprotech Cat No. AF-100–15). The cells were maintained as neurospheres and were passaged every 5–6 days using mechanical procedures. All experiments were performed with cells between passage 3 and 15 while they maintained normal karyotype.

For proliferation assays, *HDAC1*^+/+^ and *HDAC1*^fl/fl^ neurospheres were dissociated and infected with an Adenovirus-CRE-GFP or Adenovirus-GFP (MOI = 20). Cells were maintained as floating neurospheres (density at plating: 5,000 cells/cm^2^). For proliferation assays, cells were plated on polyornithine and fibronectin-coated glass coverslips (density at plating: 10,000 cells/cm^2^) in DMEM/F12-N2 with daily addition of FGF-2 and EGF. Cells were exposed to a 1-h pulse of 5′-bromo-2-deoxyuridine (BrdU, 5 μM: Boehringer-Mannheim), then fixed with 4% PFA for 25 min and immunostained.

For differentiation assays, cells were infected as described above and plated at a density of 100,000 cells/cm^2^ on polyornithine- and fibronectin-coated glass coverslips in DMEM/F12-N2 containing FGF-2 and EGF for 24 h, then plated in the absence of mitogens for 3 extra days. Cells were then fixed with 4% PFA and immunostained.

### Cell Cycle Assays

For cell cycle assays, *HDAC1*^+/+^ and *HDAC1*^fl/fl^ cells were infected and maintained as floating neurospheres as described above. Cells were collected mechanically after 5 days in culture, washed with PBS, and fixed overnight in 70% ethanol. After an additional wash, the cells were treated with RNAse A (Sigma) for 20 min at 37°C, incubated with propidium iodide (PI, final concentration 25 μg/ml) then analyzed by flow cytometry (FACS Vantage, BD) to determine the proportion of cells in each phase of the cell cycle.

### Western Blot

Immunoblotting was performed on extracts from *HDAC1*^+/+^ and *HDAC1*^fl/fl^ cells infected with an Adenovirus-CRE-GFP maintained in proliferation or differentiation conditions as described above. The membranes were probed with primary antibodies against rabbit anti-HDAC1 (1:1,000; Abcam, Cat. No. ab19845), rabbit anti-HDAC2 (1:1,000; Abcam, Cat. No. ab32117) and mouse anti-GADPH (1:1,000; Santa Cruz, Cat. No. sc-32233). The optical density of the specific protein bands was measured by densitometry using Image J to estimate the relative protein levels. The levels of HDAC1 and HDAC2 were normalized to the levels of GADPH.

### Immunostaining of Cells in Neurosphere and Adherent Cultures

After treatment with 0.3% Triton X-100/3% NGS/TBS for 1 h, cells were incubated overnight at 4°C with primary antibodies raised against: rat anti-BrdU (1:250; Accurate, Cat. No. OBT0030); anti-DCX (1:2,000; Millipore, No. AB2253), mouse anti-GFAP (1:1,000; Millipore, Cat. No. MAB360), chicken anti-GFP (1:500; AvesLab, Cat. No. GFP-1010), rabbit anti-HDAC1 (1:1,000; Abcam, Cat. No. ab19845), rabbit anti-HDAC2 (1:1,000; Abcam, Cat. No. ab32117), rabbit anti-Ki67 (1:1,000; Thermo Fisher Scientific, Cat. No. RM-9106), mouse anti-MAP2ab (1:250; Sigma, Cat. No. m1406), rabbit anti-Sox2 (1:1,000; Millipore, Cat. No. AB5603) and mouse anti-Tuj1 (1:400; Sigma, T8660). The cells were then incubated with FITC, Cy3 and Cy5 conjugated secondary antibodies (1:500; Jackson ImmunoResearch) for 2 h at room temperature and finally with DAPI, then mounted in PVA.

### Cell Counts

Images were taken using a Nikon A1R confocal microscope or a Leica confocal microscope (TCS SPE8) equipped with four laser lines (405, 488, 561, and 633 nm) under 20×. To determine the number of cells growing in adherent culture conditions that expressed a specific antigen, 10 random fields per coverslip were counted using a 20x objective. The percentage of cells positive for specific markers was calculated out of the number of GFP^+^ cells. The percentage of GFP^+^ cells was calculated out of the number of DAPI^+^ cells.

### Statistical Analysis

A two-tailed Student’s *t*-test was used to compare the mean ± standard error of the mean (SEM) values from HDAC1^+/+^ and *HDAC1*^fl/fl^ cells or HDAC1^+/+^ and *HDAC1*^fl/fl^ mice, with Welch’s correction when the *F*-test indicated significant differences between the variances of both groups. All analyses were carried out with GraphPad Prism software. The differences were considered statistically significant when *P* < 0.05.

## Results

### Expression of HDAC1 and HDAC2 in Adult Dentate Gyrus

**FIGURE 1:**
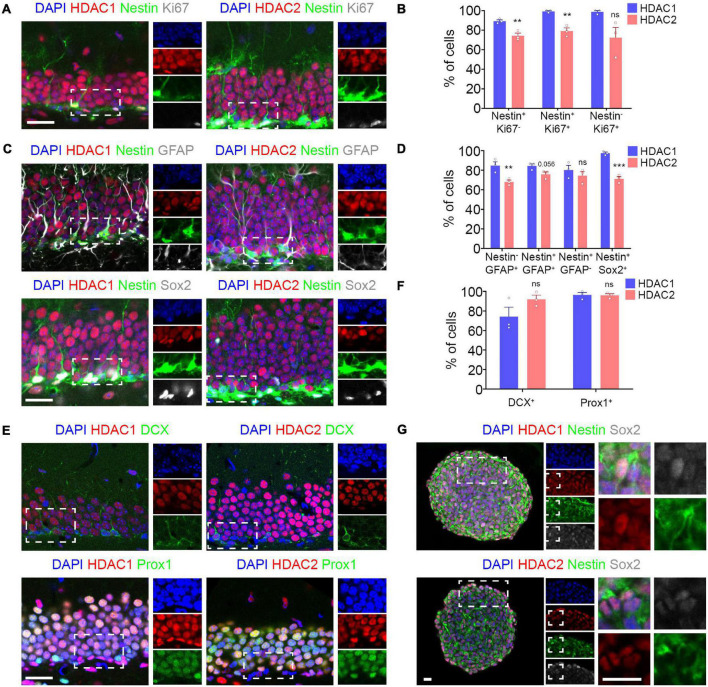
HDAC1 and HDAC2 are expressed in the dentate gyrus during adult neurogenesis. **(A)** Representative images of DG section from Nestin-GFP mice immunostained against HDAC1/HDAC2 and Ki67, and stained with DAPI. **(B)** The graph shows the percentage of quiescent stem cells (Nestin^+^ Ki67^–^), proliferative neural stem/progenitor cells (Nestin^+^ Ki67^+^) and neuronal progenitor cells (Nestin^–^ Ki67^+^) that expressed HDAC1 and HDAC2. **(C)** Representative images of DG section from Nestin-GFP mice immunostained against HDAC1/HDAC2 and GFAP and Sox2, and stained with DAPI. **(D)** The graph shows the percentage of astrocytes (Nestin^–^ GFAP^+^), type 1 cells (Nestin^+^ GFAP^+^), type 2 cells (Nestin^+^ GFAP^–^) and type 1–2 cells (Nestin^+^ Sox2^+^) that expressed HDAC1 and HDAC2. **(E)** Representative images of DG section from wild-type mice immunostained against HDAC1/HDAC2 and DCX and Prox1, and stained with DAPI. **(F)** The graph shows the percentage of immature (DCX^+^ cells) and mature granule neurons (Prox1^+^ cells) that expressed HDAC1 and HDAC2. **(G)** Representative images of HPSC neurospheres isolated from 6-week-old wild-type mice DG section immunostained against HDAC1/HDAC2 and Nestin and Sox2 and stained with DAPI. **p < 0.01, ***p < 0.001, ns = not significant. N = 3. Scale bar = 50 μm.

HDAC1 and HDAC2 have been shown to exhibit complementary expression in the embryonic and adult brain (MacDonald and Roskams, 2008; Jawerka et al., 2010; Tang et al., 2019). Moreover, they play redundant roles in the developing brain in controlling the progression from progenitor cells to mature neurons (Montgomery et al., 2009). However, it is unknown if they also have a similar role in the adult brain. To this end, we studied the expression of HDAC1 and HDAC2 at different stages during adult hippocampal neurogenesis. We found a wide expression of HDAC1 in the DG using two different antibodies (Supplementary Figure 1). As the HDAC1 expression pattern was similar, we used the antibody from abcam for further analysis. Then, we used Nestin-GFP transgenic mouse to label NSCs (Mignone et al., 2004) and colabeling with different antibodies for specific cell types to analyze the expression pattern of HDAC1 and HDAC2 during hippocampal neurogenesis (Figure 1). First, we analyzed the expression of HDAC1 and HDAC2 in quiescent stem cells and type 1 cells (Nestin^+^ Ki67^–^), proliferative neural stem/progenitor cells (Nestin^+^ Ki67^+^), and neuronal progenitor cells (Nestin^–^ Ki67^+^) (Figures 1A,B; Bonaguidi et al., 2011; Encinas et al., 2011; Hsieh, 2012). We found that HDAC1 was highly expressed in all cell types (>90%). HDAC2 was also expressed, but in a significantly lower percentage of cells. We then studied the expression of both HDACs in astrocytes (Nestin^–^ GFAP^+^), type 1 cells (Nestin^+^ GFAP^+^), type 2 cells (Nestin^+^ GFAP^–^) and type 1–2 cells (Nestin^+^ Sox2^+^) (Figures 1C,D; Bonaguidi et al., 2011; Encinas et al., 2011; Hsieh, 2012). Although HDAC1 and HDAC2 were expressed in all of them, HDAC1 was expressed in a higher percentage of cells, with the difference especially significant in Nestin^+^ Sox2^+^ cells. We next examined expression of both HDACs in immature (DCX^+^ cells) and mature granule neurons (Prox1^+^ cells) (Figures 1E,F). HDAC1 was observed in a lower percentage of DCX^+^ cells than HDAC2 (difference not significant). However, both HDACs were found in a similar percentage in Prox1^+^ cells (∼96%). Finally, we found HDAC1 and HDAC2 expression in hippocampal NSCs *in vitro* (Figure 1G). Our results demonstrated that although HDAC1 and HDAC2 are both expressed in all cellular stages during hippocampal neurogenesis, more neural stem/progenitor cells express HDAC1 compared to HDAC2. In contrast, HDAC2 is found in a higher percentage in immature neurons.

### HDAC1 Does Not Affect Proliferation of Hippocampal Neural Stem Cells

**FIGURE 2:**
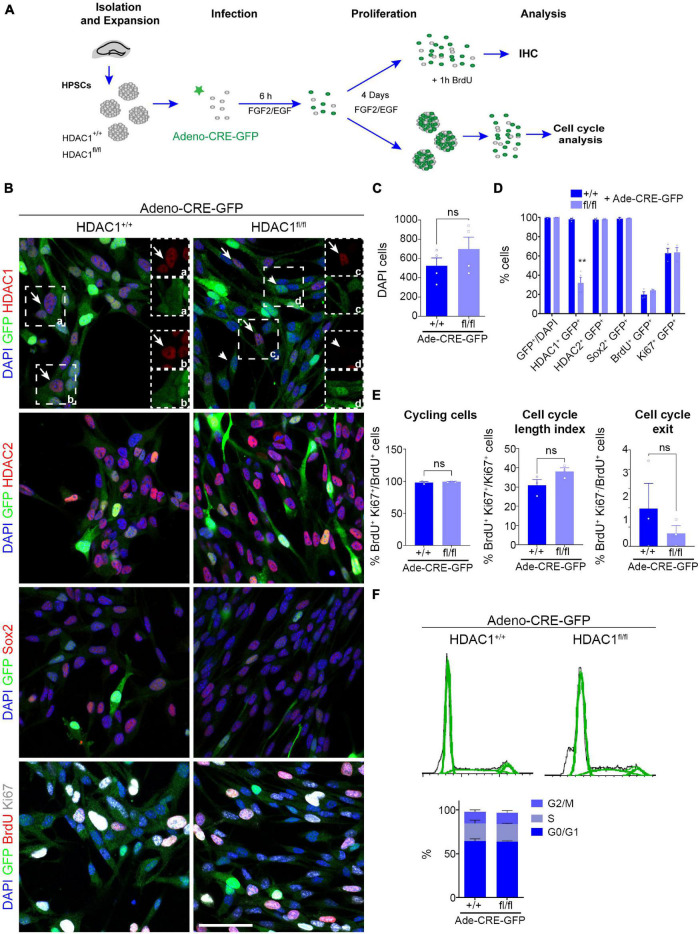
HDAC1 deletion does not affect HPSC proliferation *in vitro*. **(A)** Experimental design of expansion, infection and maintenance of HPSCs in proliferation condition. After infection cells were kept in proliferation condition (plus EGF and FGF2) for 4 days attached to coverslips for immunostaining analysis or as neurospheres for cell cycle analysis by flow cytometry. **(B)** Representative images of HDAC1^+/+^ and HDAC1^fl/fl^ HPSCs infected with adeno-Cre-virus immunostained against GFP, HDAC1, HDAC2, Sox2, BrdU and Ki67, and stained with DAPI. **(C)** The graph shows the number of DAPI cells in 10 random fields in HDAC1^+/+^ and HDAC1^fl/fl^ cells infected with an adeno-Cre-GFP virus. **(D)** The graph shows the percentage of GFP^+^ cells out of the DAPI cells and the percentage of GFP^+^ cells expressing HDAC1, HDAC2, Sox2, BrdU and Ki67. **(E)** The graphs show the percentage of cycling cells (BrdU^+^ Ki67^+^/BrdU^+^ cells), cell cycle length index (BrdU^+^ Ki67^+^/Ki67^+^ cells) and the percentage of cell cycle exit (BrdU^+^ Ki67^–^/BrdU^+^ cells). **(F)** The graphs show representative cell cycle profiles from HDAC1^+/+^ and HDAC1^fl/fl^ cells infected with an adeno-Cre virus and percentage of cells in each phase of the cell cycle. Arrows point out HDAC1^+^ cells and arrowheads HDAC1^–^ cells. **p < 0.01, ns = not significant. *N* = 4. Scale bar = 50.

The higher expression of HDAC1 in neural stem/progenitor cells prompted us to study the role of HDAC1 in cell proliferation *in vitro* (Figure 2). We isolated HPSCs from 6-week old HDAC1^+/+^ and HDAC1^fl/fl^ littermates and grew them as neurospheres (Figure 2A). We infected them using an adeno-Cre-GFP virus to knock-out HDAC1 and we maintained them in proliferation conditions for 4 days. We found no differences in the number of cells after 4 days *in vitro* (DIV, Figures 2B,C). Moreover, almost 100% of the cells were GFP^+^ in both conditions (Figures 2B,D). Most of the cells were HDAC1^+^ in HDAC1^+/+^ cells. In contrast, we found a significant reduction in the percentage of HDAC1^+^ cells in the HDAC1^fl/fl^ cells infected with the adeno-Cre-GFP virus. Although we found some HDAC1^+^ cells in HDAC1^fl/fl^ cells, the expression level was lower than in HDAC1^+/+^ cells (Figure 2B). A similar percentage of cells expressed HDAC2 in both conditions. Similar results were found by western blot (Supplementary Figures 2A,B). To confirm that HDAC1 reduction in HDAC1^fl/fl^ cells is specific to Cre expression, we also infected both HDAC1^+/+^ and HDAC1^fl/fl^ cells with an adeno-GFP virus that did not contain the *Cre* gene; we found no difference in the percentage of cells expressing HDAC1 or HDAC2 in any of those conditions (Supplementary Figure 3). We then analyzed the percentage of cells expressing Sox2, BrdU (after a 1-h pulse), and Ki67; we did not see any difference between HDAC1^+/+^ and HDAC1^fl/fl^ cells infected with Cre virus. Moreover, we found no differences in the percentage of cells cycling or exiting the cell cycle (Figures 2B,E). We also did detect no appreciable number of Tuj1^+^ or Casp3^+^ cells under either condition (data not shown). We had similar results after dissociating the neurospheres and analyzing the percentage of cells in each phase of the cell cycle by flow cytometry (Figure 2F). Altogether, our results showed that HDAC1 depletion does not affect cell proliferation in HPSCs *in vitro*.

### HDAC1 Deletion Compromises Neuronal Differentiation *in vitro*

**FIGURE 3:**
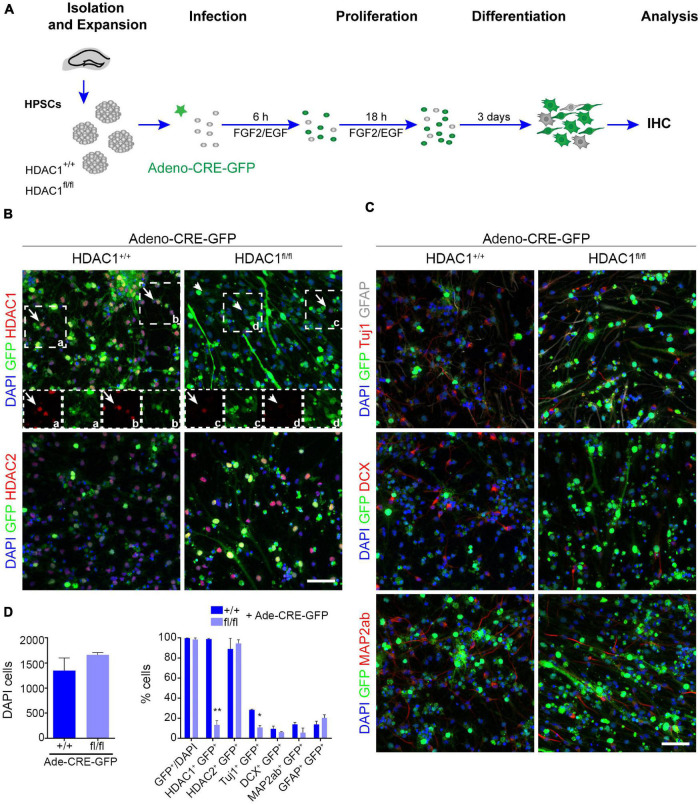
HDAC1 affects HPSC differentiation *in vitro*. **(A)** Experimental design of expansion, infection and maintenance of HPSCs in differentiation condition. After infection cells were kept in differentiation condition (EGF and FGF2 were removed 24 h after infection) for 4 days. **(B)** Representative images of HDAC1^+/+^ and HDAC1^fl/fl^ HPSCs infected with adeno-Cre-virus immunostained against GFP, HDAC1, HDAC2, and stained with DAPI. **(C)** Representative images of HDAC1^+/+^ and HDAC1^fl/fl^ HPSCs infected with adeno-Cre-virus immunostained against GFP, Tuj1, GFAP, DCX and MAP2ab, and stained with DAPI. **(D)** The graphs show the number of DAPI cells in 10 random fields in HDAC1^+/+^ and HDAC1^fl/fl^ cells infected with an adeno-Cre-GFP virus and the percentage of GFP^+^ cells out of the DAPI cells and the percentage of GFP^+^ cells expressing HDAC1, HDAC2, Tuj1, DCX, MAP2ab, and GFAP. *p < 0.05, **p < 0.01, ns = not significant. *N* = 2. Scale bar = 50.

Valproic acid, a HDAC inhibitor, has been shown to affect cell proliferation and differentiation in neural progenitor cells *in vitro* and promotes aberrant hippocampal neurogenesis after prenatal exposure (Hsieh et al., 2004; Sakai et al., 2018). Nevertheless, we detected no change in proliferation after HDAC1 was knocked out in HPSCs HDAC1 by viral infection. Thus, we decided to evaluate the role of HDAC1 in differentiation conditions. HDAC1^+/+^ and HDAC1^fl/fl^ cells were infected with Cre virus for 24h, and were maintained without growth factors for 3 days to induce differentiation (Figure 3A). We observed no difference in the number of cells after 3 DIV (Figures 3B–D). Similar to the results found in proliferation conditions, almost all the cells expressed GFP under both conditions, but only HDAC1^fl/fl^ cells showed a significant reduction of HDAC1 expression (Figures 3B,D). HDAC1 reduction was also confirmed by western blot (Supplementary Figures 2C,D). Interestingly, we observed a significant decrease of Tuj1^+^ cells after HDAC1 deletion (Figures 3C,D). We also found a decrease of DCX^+^ and MAP2ab^+^ cells in HDAC1^fl/fl^ cells. In contrast, GFAP^+^ cells slightly increased after HDAC1 loss. These results suggest that HDAC1 plays a role in hippocampal neuronal differentiation.

### HDAC1 Expression Was Not Reduced *in vivo*

**FIGURE 4:**
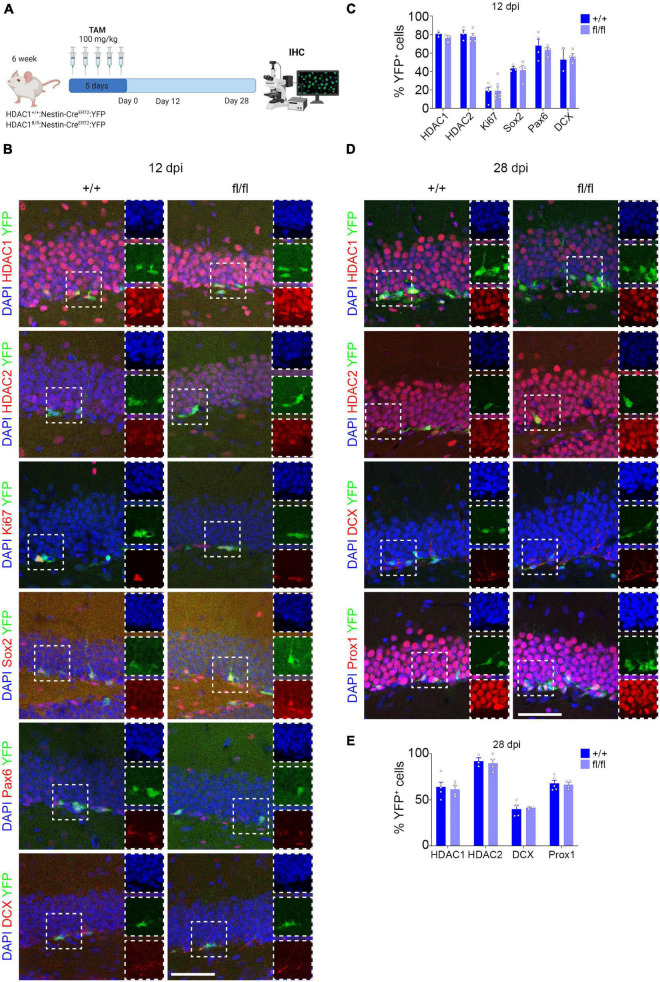
Conditional HDAC1 deletion in neural stem cells *in vivo*. **(A)** Experimental design of HDAC1^+/+^ and HDAC1^fl/fl^:Nestin-CRE^ERT2^:YFP mice after TAM injections for 5 days. Mice were perfused and analyzed by IHC 12 and 28 dpi. **(B)** Representative images of HDAC1^+/+^ and HDAC1^fl/fl^ mice after 12 dpi immunostained against YFP, HDAC1, HDAC2, Ki67, Sox2, Pax6, and DCX; and stained with DAPI. **(C)** The graph shows the percentage of HDAC1^+^, HDAC2^+^, Ki67^+^, Sox2^+^, Pax6^+^ and DCX^+^ out of the YFP^+^ cells at 12 dpi. **(D)** Representative images of HDAC1^+/+^ and HDAC1^fl/fl^ mice after 28 dpi immunostained against YFP, HDAC1, HDAC2, DCX and Prox1; and stained with DAPI. **(E)** The graph shows the percentage of HDAC1^+^, HDAC2^+^, DCX^+^ and Prox1^+^ out of the YFP^+^ cells at 28 dpi. dpi = days post inyection, IHC = immunohistochemistry, TAM = tamoxifen. *N* = 2–5. Scale bar = 50.

The decrease in Tuj1^+^ cells *in vitro* due to the loss of HDAC1 prompted us to investigate the role of HDAC1 *in vivo* during adult hippocampal neurogenesis. To this end, we crossed HDAC1 floxed mice to Nestin-CRE^ERT2^ to selectively knock-out HDAC1 in NSCs after TAM injection (Lagace et al., 2007). We also crossed the mice to the ROSA26-YFP reporter line to visualize the cells (Srinivas et al., 2001) that expressed CRE after TAM injection and would thereby indicate where HDAC1 was knocked-out. Mice were perfused after 12 dpi to evaluate the effect of the loss of HDAC1 in proliferation and progenitor cells, and after 28 dpi to study its effect on neuronal differentiation (Figure 4A). We first analyzed the percentage of HDAC1^+^, HDAC2^+^, Ki67^+^ (proliferative cells) (Tanaka et al., 2011), Sox2^+^ and Pax6^+^ cells (neural stem/progenitor cells) (Suh et al., 2007; Sansom et al., 2009) and DCX^+^ cells (immature neurons) (Bracko et al., 2012) at 12 dpi (Figures 4B,C). Surprisingly, the percentage of YFP^+^ cells expressing HDAC1 (∼80%) was similar in HDAC1^+/+^ and HDAC1^fl/fl^ mice. In addition, we measured the fluorescent intensity of HDAC1 in HDAC1^+/+^ and HDAC1^fl/fl^ mice, but we saw no differences between the two genotypes (Supplementary Figure 4A). Neither the percentage nor the total number of cells expressing any of markers was different in either genotype (Figure 4C and Supplementary Figure 4B). It could be possible that 12 dpi was not enough to remove all HDAC1 protein in the cells, even if the gene were deleted properly, so we analyzed the percentage of HDAC1^+^, HDAC2^+^, DCX^+^ cells (immature neurons) and Prox1^+^ cells (mature granule neurons) (Iwano et al., 2012) at 28 dpi (Figures 4D,E). Consistent with our data on HDAC1 expression, we found a lower percentage of YFP^+^ cells that expressed HDAC1 at 28 dpi (∼60%) than at 12 dpi. Nevertheless, the percentage and number of HDAC1^+^ cells and the fluorescent intensity were similar in HDAC1^+/+^ and HDAC1^fl/fl^ mice (Figure 4E and Supplementary Figures 4C,D). Moreover, we saw no differences in any of the other markers (Figure 4E and Supplementary Figure 4D). These data showed that 5 days of TAM injection was not sufficient to reduce the expression of HDAC1 in NSCs *in vivo*.

Altogether, our data suggest that HDAC1 controls HPSC differentiation without affecting cell proliferation *in vitro*. However, further investigations would be needed to test that effect *in vivo* during adult neurogenesis.

## Discussion

Histone deacetylases, which remove acetyl groups from histones to inhibit gene expression (D’Mello, 2020), have been shown to play a key role during brain development (Hsieh et al., 2004; Montgomery et al., 2009; Jawerka et al., 2010; Lee et al., 2016; Sakai et al., 2018; Tang et al., 2019). HDAC inhibitors have been used to treat neurological disorders such as epilepsy, even during pregnancy (Sakai et al., 2018). However, most of these inhibitors are not specific in their actions; they affect multiple genes including some that encode non-histone proteins. Some studies have been undertaken to understand the role of individual HDACs, in order to develop more specific treatments, but the effects of some HDACs during brain development— especially HDAC1—are still unknown.

Our results show that HDAC1 expression is critical for proper differentiation of HPSC *in vitro*. We also found that HDAC1 deletion is sufficient to compromise neuronal differentiation, and that HDAC2 cannot compensate for loss of HDAC1.

### HDAC1 and HDAC2 Expression in the Adult Hippocampus

HDAC1 and HDAC2 are highly expressed and overlap in neuroepithelial cells and NSCs during early development (Tang et al., 2019). Other studies have shown that postnatally, HDAC1 is expressed mainly in astrocytes, whereas HDAC2 is found in neurons (MacDonald and Roskams, 2008; Jawerka et al., 2010). In the adult HP, HDAC1 is highly expressed in NSCs, while HDAC2 expression is higher in granule neurons in the dentate gyrus (DG) of the HP (Jawerka et al., 2010; Foti et al., 2013). However, our data showed that HDAC1 and HDAC2 expression overlap in a high percentage of cells in all the cellular stages during adult hippocampal neurogenesis. We found that HDAC1 is expressed in a higher percentage of neural stem and progenitor cells in the HP than is HDAC2. The differences between the results presented by Jawerka et al. (2010) and ours could be due to the age of the mice: Jawerka et al. (2010) used 3-month old mice while we used 6-week-old mice. It is possible that while HDAC1 and HDAC2 expression overlap during brain development, their expression is more restricted postnatally. Alternatively, the differences could be due to the use of different antibodies, although we found a similar expression pattern using two different antibodies. Our findings could be consistent with an intermediate stage between the expression of both HDACs embryonically and in adult. More studies would be needed to clarify the expression of HDAC1 and HDAC2 over time.

### HDAC1 Loss Affects Neuronal Differentiation *in vitro*

Valproic acid is a HDAC inhibitor prescribed to control seizure activity, even in pregnant women. Moreover, VPA has been shown to promote both neurogenesis and gliogenesis (Hsieh et al., 2004; Lee et al., 2016). In addition, VPA increases seizure susceptibility in adult offspring due to aberrant hippocampal neurogenesis (Sakai et al., 2018). These results highlight the importance of understand how HDACs affect neural differentiation and their long-term consequences, to identify more specific and better treatments for neurodevelopment disorders. To this end, it has been shown that HDAC2 is necessary for the formation of new neurons in the adult DG (Jawerka et al., 2010). On the other hand, HDAC3 deletion promotes a reduction of neural stem cell proliferation and neuronal differentiation in the adult HP (Jiang and Hsieh, 2014). Our results showed HDAC1 reduction compromises the capacity of HPSCs to produce neurons *in vitro*. It might look surprising that, although VPA promotes neuronal differentiation in rat neural progenitors (Hsieh et al., 2004), HDAC1 deletion decrease neuronal production in NSCs. One explanation could be that the neural progenitor cells used by Hsieh et al. (2004) were at a more differentiated stages than the primary NSCs used in this study, and therefore their response to HDAC1 inhibition was different. Another possibility is that the reduction of neuronal differentiation by inhibition of HDAC1 using VPA could be mediated by the inhibition of other HDACs. Furthermore, our results also showed that the deletion of HDAC1 is sufficient to compromise neuronal differentiation and HDAC2 expression cannot compensate for HDAC1 absence. However, during embryonic development both HDAC1 and HDAC2 have complementary roles (Montgomery et al., 2007). More studies using specific HDAC inhibitor or transgenic mice would be needed to clarify the role of each HDAC during brain development, especially in adult where the expression of each HDAC is more restricted.

### Deletion of HDAC1 *in vivo*

The use of transgenic mice has been incredibly useful to study the role of a variety of genes during development (Asai et al., 2012; Nieto-Estevez et al., 2016). Moreover, conditional knockout mice allow control the gene expression in a cellular and time specific manner (Kos, 2004; Montgomery et al., 2009; Jawerka et al., 2010; Jiang and Hsieh, 2014; Nieto-Estevez et al., 2016). In this study, we used Nestin-CRE^ERT2^ mice to drive the expression of CRE in NSCs when TAM was administrated intraperitoneally. However, we could not detect a reduction in the expression of HDAC1 at 12 or 28 dpi, either in the number of cells or in the intensity of fluorescence. CRE expression was probably sufficient to promote the expression of YFP in the DG and in the subventricular zone (data not shown), while the level of CRE expression needed to excise the loxP sites on HDAC1 gene was too low. In previous works from the lab, 150 mg/kg of TAM effectively reduced the expression of REST or HDAC3 (Gao et al., 2011; Jiang and Hsieh, 2014). However, that concentration in HDAC1:Nestin-CRE^ERT2^:YFP mice proved fatal during the 5 days of TAM injection or a few days after the last injection (data not shown). We thus reduced the TAM dose to 100 mg/kg, which has been used in other studies (Mori et al., 2006; Jawerka et al., 2010). Different strains of mice might have a different tolerance to TAM administration, so higher doses could be toxic in certain strains. Another explanation could be that we effectively excised HDAC1 gene but the protein is unusually stable and takes longer to be completely removed from the cells. In addition, *in situ* hybridization or RNA-scope techniques might be necessary to determine HDAC1 reduction at mRNA level. Alternativity, PCR of genomic DNA from YFP^+^ cells would confirm HDAC1 excision. Unfortunately, our data did not provide evidence on whether HDAC1 plays a role during hippocampal neurogenesis *in vivo*. Successfully deleting HDAC1 *in vivo* may require other strains of mice or other techniques, such as viral injection.

Altogether, our results show that HDAC1 plays a role during neuronal differentiation of hippocampal stem cells and highlights the importance of fully understanding the role of specific HDACs during brain development, to create better treatments for neurodevelopmental diseases.

## Data Availability Statement

The original contributions presented in the study are included in the article/Supplementary Material, further inquiries can be directed to the corresponding author/s.

## Ethics Statement

The animal study was reviewed and approved by Institutional Animal Care and Use Committee at the University of Texas Southwestern Medical Center (UTSW) and the University of Texas at San Antonio (UTSA).

## Author Contributions

VN-E: design, collection and assembly of data, data analysis and interpretation, and manuscript writing. GC, AA, MC, RK, and JZ: collection and assembly of the data. JH: conception and design, data analysis and interpretation, financial support, manuscript editing, and final approval of manuscript. All authors contributed to the article and approved the submitted version.

## Conflict of Interest

The authors declare that the research was conducted in the absence of any commercial or financial relationships that could be construed as a potential conflict of interest.

## Publisher’s Note

All claims expressed in this article are solely those of the authors and do not necessarily represent those of their affiliated organizations, or those of the publisher, the editors and the reviewers. Any product that may be evaluated in this article, or claim that may be made by its manufacturer, is not guaranteed or endorsed by the publisher.

## Supplementary Material

Supplementary Figure 1 Analysis of HDAC1 expression *in vivo*. **(A)** Representative images of DG section from adult mice immunostained against HDAC1 (abcam), and stained with DAPI. **(B)** Representative images of DG section from adult mice immunostained against HDAC1 (Cell Signaling), and stained with DAPI. *N* = 3. Scale bar = 50.

Supplementary Figure 2 Analysis of HDAC1 expression by western blot. **(A)** Experimental design of expansion, infection and maintenance of HPSCs in proliferation condition. After infection cells were kept in proliferation condition (plus EGF and FGF2) for 4 days proteins were extracted and protein levels were measured by western blot. **(B)** The graph shows the relative HDAC1 and HDAC2 levels normalized to GADPH in HDAC1^+/+^ and HDAC1^fl/fl^ HPSCs in proliferative condition. The images show HDAC1, HDAC2 and GADPH levels. **(C)** Experimental design of expansion, infection and maintenance of HPSCs in differentiation condition. After infection cells were kept in proliferation condition (plus EGF and FGF2) for 24 h and in differentiation condition for 3 days. Then proteins were extracted and protein levels were measured by western blot. **(D)** The graph shows the relative HDAC1 and HDAC2 levels normalized to GADPH in HDAC1^+/+^ and HDAC1^fl/fl^ HPSCs in differentiation condition. The images show HDAC1, HDAC2 and GADPH levels. *N* = 2.

Supplementary Figure 3 HDAC1 expression after infection with an adeno-GFP virus. HPSCs from HDAC1^+/+^ and HDAC1^fl/fl^ mice were expanded, infected with an adeno-GFP virus and maintenance in proliferation condition. After infection cells were kept in proliferation condition (plus EGF and FGF2) for 4 days attached to coverslips for immunostaining analysis. **(A)** Representative images of HDAC1^+/+^ and HDAC1^fl/fl^ HPSCs infected with adeno-GFP-virus immunostained against GFP, HDAC1, HDAC2, and stained with DAPI. **(B)** The graphs show the percentage of GFP^+^ cells expressing HDAC1 and HDAC2. ***p* < 0.01, ns = not significant. *N* = 2–4. Scale bar = 50.

Supplementary Figure 4 Conditional HDAC1 deletion in neural stem cells *in vivo*. **(A)** The graph shows the mean of the HDAC1 fluorescent intensity in YFP^+^ cells at 12 dpi. **(B)** The graph shows the total number of YFP^+^ and the total number of HDAC1^+^, HDAC2^+^, Ki67^+^, Sox2^+^, Pax6^+^, and DCX^+^ cells out of the YFP^+^ cells at 12 dpi. **(C)** The graph shows the mean of the HDAC1 fluorescent intensity in YFP^+^ cells at 28 dpi. **(D)** The graph shows the total number of YFP^+^ and the total number of HDAC1^+^, HDAC2^+^, DCX^+^, and Prox1^+^ cells out of the YFP^+^ cells at 28 dpi. dpi, days post injection. *N* = 2–5.
