# Supplementary figures and images for: HDAC1 Regulates Neuronal Differentiation
Source: Front Mol Neurosci. 2022 Jan 12;14:815808. doi: 10.3389/fnmol.2021.815808 (PMC8789757; doi:10.3389/fnmol.2021.815808)

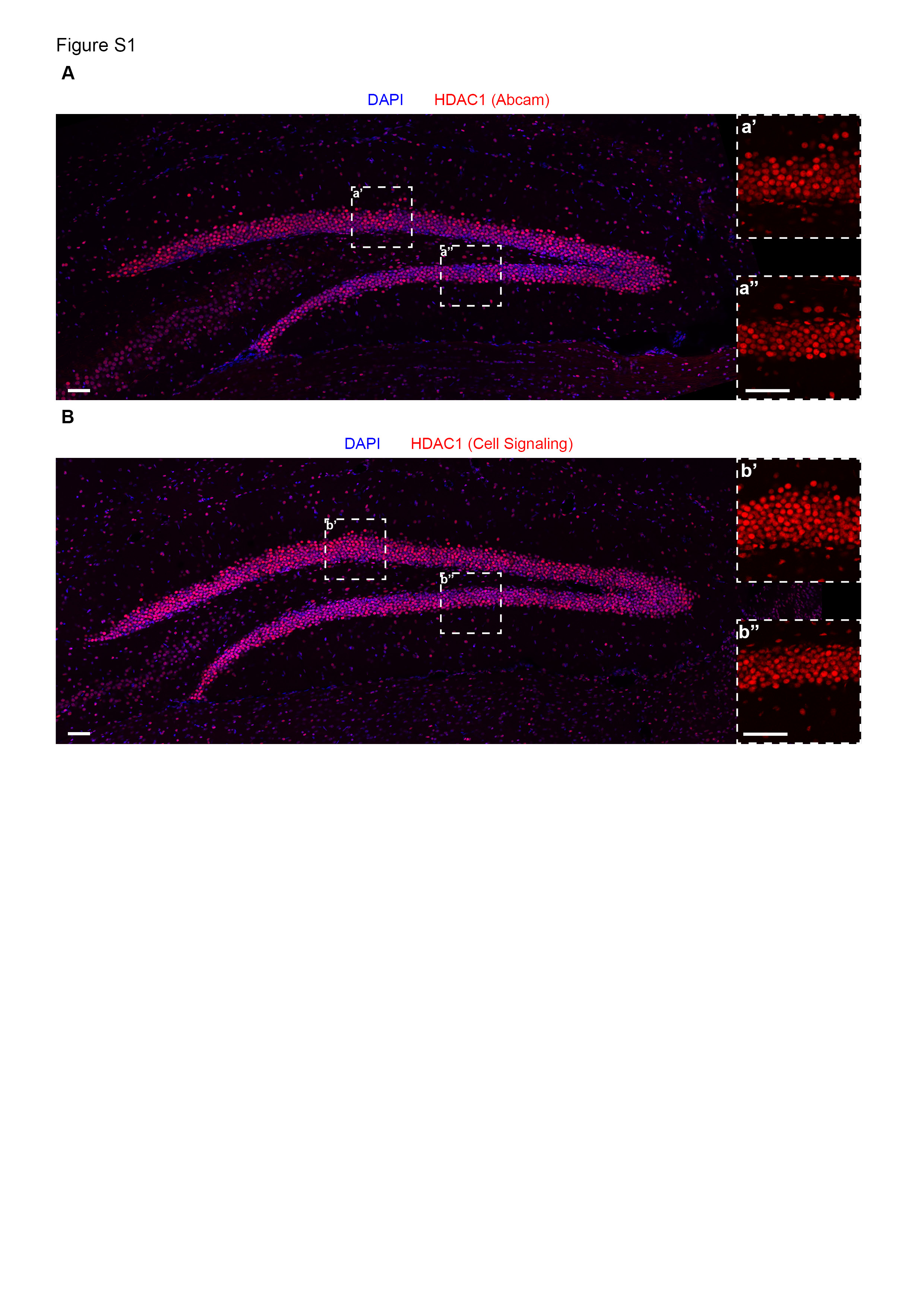

Supplement: Supplementary Figure 1 — Analysis of HDAC1 expression in vivo. (A) Representative images of DG section from adult mice immunostained against HDAC1 (abcam), and stained with DAPI. (B) Representative images of DG section from adult mice immunostained against HDAC1 (Cell Signaling), and stained with DAPI. N = 3. Scale bar = 50. [file Image_1.JPEG]

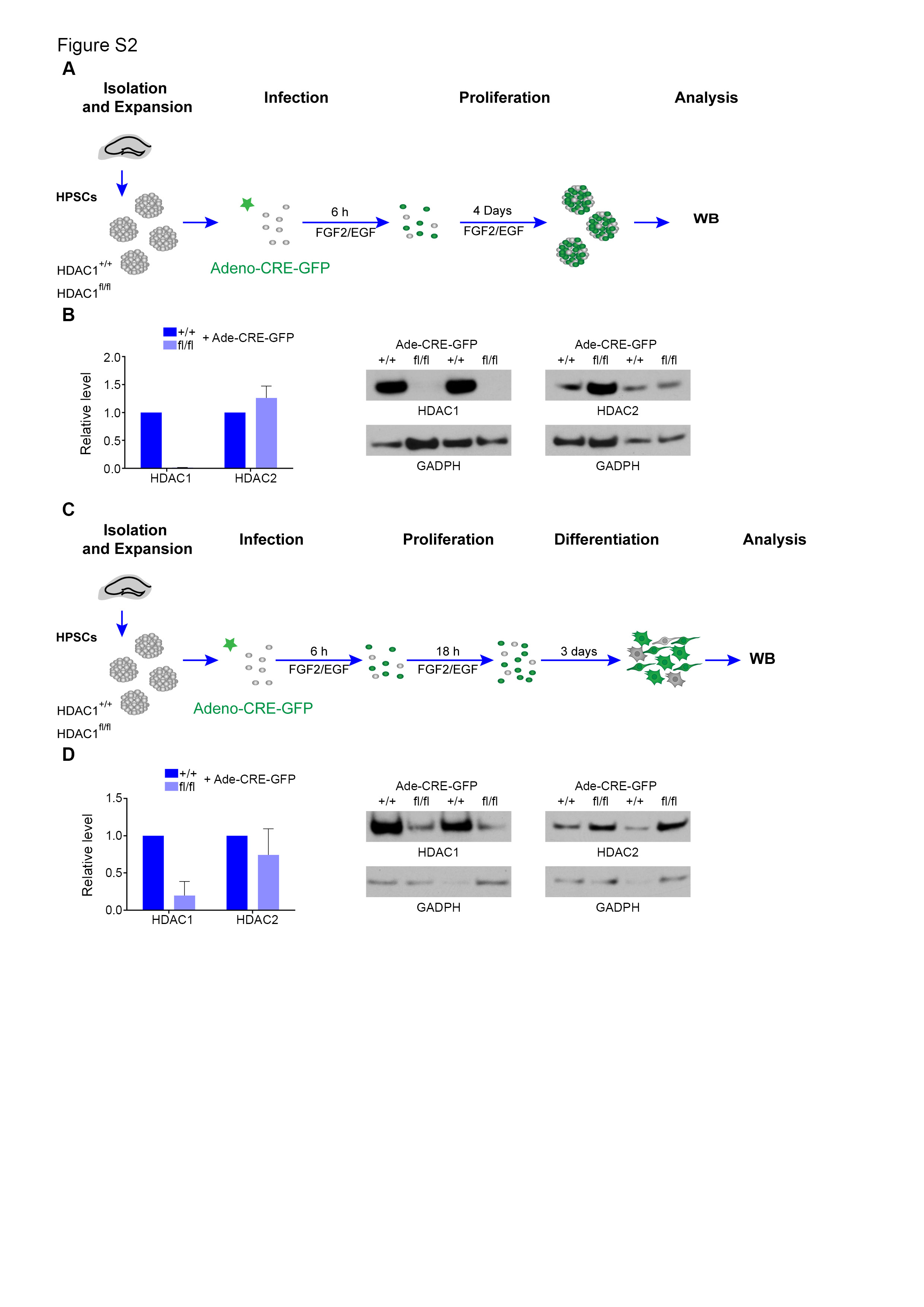

Supplement: Supplementary Figure 2 — Analysis of HDAC1 expression by western blot. (A) Experimental design of expansion, infection and maintenance of HPSCs in proliferation condition. After infection cells were kept in proliferation condition (plus EGF and FGF2) for 4 days proteins were extracted and protein levels were measured by western blot. (B) The graph shows the relative HDAC1 and HDAC2 levels normalized to GADPH in HDAC1+/+ and HDAC1fl/fl HPSCs in proliferative condition. The images show HDAC1, HDAC2 and GADPH levels. (C) Experimental design of expansion, infection and maintenance of HPSCs in differentiation condition. After infection cells were kept in proliferation condition (plus EGF and FGF2) for 24 h and in differentiation condition for 3 days. Then proteins were extracted and protein levels were measured by western blot. (D) The graph shows the relative HDAC1 and HDAC2 levels normalized to GADPH in HDAC1+/+ and HDAC1fl/fl HPSCs in differentiation condition. The images show HDAC1, HDAC2 and GADPH levels. N = 2. [file Image_2.JPEG]

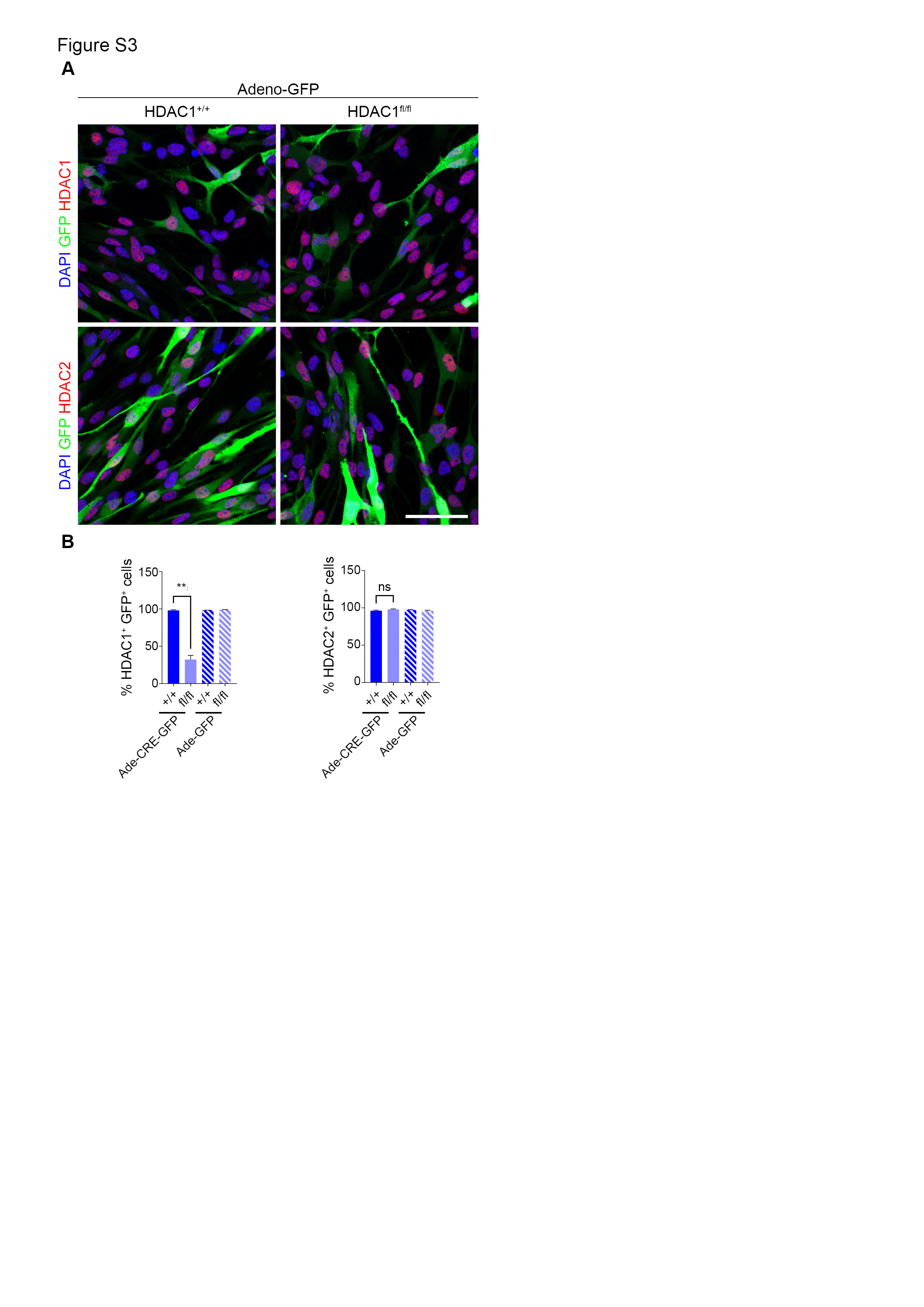

Supplement: Supplementary Figure 3 — HDAC1 expression after infection with an adeno-GFP virus. HPSCs from HDAC1+/+ and HDAC1fl/fl mice were expanded, infected with an adeno-GFP virus and maintenance in proliferation condition. After infection cells were kept in proliferation condition (plus EGF and FGF2) for 4 days attached to coverslips for immunostaining analysis. (A) Representative images of HDAC1+/+ and HDAC1fl/fl HPSCs infected with adeno-GFP-virus immunostained against GFP, HDAC1, HDAC2, and stained with DAPI. (B) The graphs show the percentage of GFP+ cells expressing HDAC1 and HDAC2. **p < 0.01, ns = not significant. N = 2–4. Scale bar = 50. [file Image_3.JPEG]

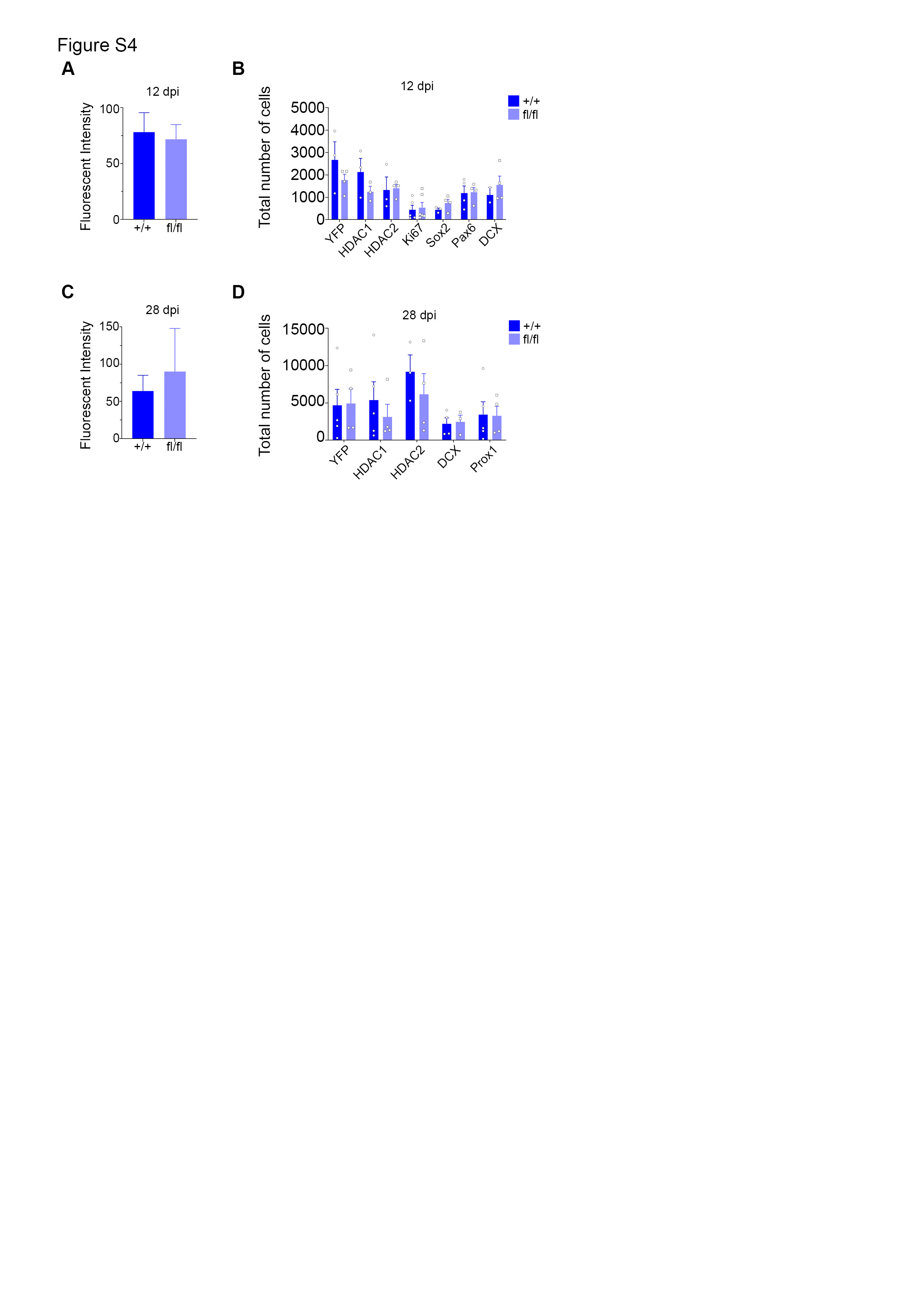

Supplement: Supplementary Figure 4 — Conditional HDAC1 deletion in neural stem cells in vivo. (A) The graph shows the mean of the HDAC1 fluorescent intensity in YFP+ cells at 12 dpi. (B) The graph shows the total number of YFP+ and the total number of HDAC1+, HDAC2+, Ki67+, Sox2+, Pax6+, and DCX+ cells out of the YFP+ cells at 12 dpi. (C) The graph shows the mean of the HDAC1 fluorescent intensity in YFP+ cells at 28 dpi. (D) The graph shows the total number of YFP+ and the total number of HDAC1+, HDAC2+, DCX+, and Prox1+ cells out of the YFP+ cells at 28 dpi. dpi, days post injection. N = 2–5. [file Image_4.JPEG]
